# Chicago Medical Society

**Published:** 1881-05

**Authors:** 


### ARTICLE VII.

### CHICAGO MEDICAL SOCIETY—THIRTY-FIRST ANNUAL MEETING, HELD APRIL 4, 1881. Dr. R. G. Bogue, President, in the chair.

This was one of the largest meetings of the society since its foundation. There were about forty members present, and twenty-five visitors by invitation. The names of Drs. Chas. C. Sparey, graduate of Chicago Medical College, and W. W. Allport, graduate of Rush, and that of Dr. H. D. Valin, graduate of the University of Vermont Medical College, were presented for membership and referred to the censors. The President welcomed the numerous visitors in a few words, and the Secretary, Dr. L. H. Montgomery, read the annual report of the society.

There were seventeen meetings held in the course of the year. None had taken place during the months of July, August or September, except one held on the occasion of the death of the late Treasurer, Dr. F. H. Davis. Number of members attending during the year, two hundred and seventy-eight; of visitors, seventy-four. The members of the society numbered one hundred and eighty-one at the beginning of the year. There were ten new members elected, and the society lost five members, leaving a total of one hundred and eighty-six. Of the five members lost, one had died, one left the city, another had never claimed membership, one retired of his own accord, and another refused to pay the annual fee. The Secretary had received $8.00 and expended $7.00 for postage and for a convenient record book for the society. The following papers had been read at the various meetings by the following members: Mastoid or Post Aural Abscess opening into the External Auditory Canal; Dr. F. C. Hotz. Causes of Epilepsy, with Photographs of Atrophy of one-half the Brain; Dr. S. R. Millard. Some Peculiarities of the Cerebral Circulation; Dr. J. S. Jewell. Lost Balance and Surface Treatment; Dr. G. Sprague. Diphtheria, with a History of Fifty Inoculated Cases; Dr. J. T. Everett. Atresia of the Genital Passages of Women; Dr. E. W. Jenks. Recent Progress in Brain Studies (illustrated with models); S. V. Clevenger. Woorara (Curare), its Analysis and Employment in Fifty-four Cases of Epilepsy; Dr. F. E. Waxham. Report of a Case of Meningo-Encephalitis; Dr. H. M. Bannister. A Review of the Progress of Medical Education in Chicago, with some Suggestions for its Advancement; Dr. E. Ingals. Genital Irritation and Hygiene of the Genital Organs of Young Children; Dr. R. Park. Expert Testimony, and the Microscopical Examination of Blood; Dr. R. H. Piper. Abscess in the Lower Part of the Tibia; Fibroid Tumor in Rectus Abdominis; Radical Cure of Varicocele; Dr. R. G. Bogue. Local Treatment of Chronic Gonorrhœa, by means of the Endoscope; Drs. C.

Fenger and A. Hinde. Some Sources of Error in Measuring with the Microscope; Dr. Lester Curtis. Report of a Gynæcological Case and Prolonged Gestation; Drs. Mary H. Thompson and John Bartlett. Mechanism of the Great Abscesses; Dr. E. Andrews. Health Aspect of Water Supplies; R. S. G. Paton, PH. D. The Gynæcological Chairs; W. W. Copp. Remarks on the Norton Attachment for Waste Pipes and Obstruction of Sewer Gas; Dr. R. Tilley.

None but original articles which had not been read in any other society before, were accepted. The following pamphlets were received: The Chicago Medical Review for 1880–1881; the Annual Transactions of the State Medical Society of Arkansas; the Annual Transactions of the State Medical Society of Tennessee; the Official Register of Physicians and Midwives of the State of Illinois; the Annual Report of the Illinois State Board of Health. The foundation of other scientific societies seemed to have diminished the interest placed in the society by its members; however, this last year had been more prosperous than any previous one.

Before retiring, the Secretary advocated a more thorough organization, a larger attendance of the members; that each member should be appointed to read an essay in his turn after a convenient time for preparation; that a more convenient place of meeting be secured, such as the Academy of Sciences, where there is an easy access to pathological specimens in the Museum; and an annual banquet be held in the manner of the alumni of colleges. He proposed that the initiation fee be increased to $2.00, and the annual fee to $3.00, to provide for the publication of the year's proceedings. He also believed that the usual announcement of meetings on postal cards might be discontinued, and notices sent to the daily papers.

The Treasurer next made his report. On his being elected to this office last summer, Dr. E. Fletcher Ingals found $17.00 in the treasury. He collected $211.00 in January alone. He paid out $54.93, and there is $173.67 left in the treasury. Both reports were unanimously accepted, $25.00 were presented to the Secretary, and $50.00 to the Grand Pacific Hotel, where the meetings take place.

The society proceeded to elect its officers for the coming year. Dr. E. Ingals was elected President; Dr. Mary H. Thompson, Vice President; Dr. L. H. Montgomery was re-elected Secretary; and Dr. E. Fletcher Ingals was re-elected Treasurer. The choice of a Committee of Censors was entrusted to the new President, who will name them at the next meeting.

A motion was also passed that the Secretary issue credentials to any member applying for them, to attend the annual meeting of the American Medical Association, at Richmond, Virginia, May, 3, 1881, as delegates.

The paper of the evening was read by Dr. Oscar C. DeWolf, Health Officer. It is a very valuable paper on the History and Nature of Vaccine Virus, and is published in this number of the JOURNAL. Dr. DeWolf also furnished the following interesting notes: The Health Department vaccinated 21,325 people last year. Of this number 8325 were primary; and among these ninety-three per cent. took, while in those who had been previously vaccinated, eighty-one per cent. was efficient. The daily attendance of school children in Chicago is 57,000. In 560 cases of vaccination among these, twenty of them exhibited some unusual symptoms. There were fifteen cases of dermatitis, so-called erysipelas; two or three cases of axillary abscesses (adenitis); three cases had suppurative inflammation and sloughing. He saw twenty-five cases of this kind in four years, and believes that the patient enjoys the same immunity from variola as in common cases of vaccination. One such case, a nursling, was taken to the small-pox hospital with its mother, for some peculiar reasons, and was there a week without becoming infected. Dr. DeWolf has also observed three cases of diphtheritic membranes forming over the point of inoculation. Re-vaccination is not appreciated by the people, and he calls on every physician in the city to bring his influence to bear on that point, for he is well aware that the few centers where small pox is now discovered, are precisely those where vaccination has received most opposition from an ignorant class, mostly composed of foreigners, who perpetuate the disease in their midst.

